# Xiaoyaosan Alleviates Hippocampal Glutamate-Induced Toxicity in the CUMS Rats via NR2B and PI3K/Akt Signaling Pathway

**DOI:** 10.3389/fphar.2021.586788

**Published:** 2021-04-12

**Authors:** Xue-Ming Zhou, Chen-Yue Liu, Yue-Yun Liu, Qing-Yu Ma, Xin Zhao, You-Ming Jiang, Xiao-Juan Li, Jia-Xu Chen

**Affiliations:** ^1^ School of Traditional Chinese Medicine, Beijing University of Chinese Medicine, Beijing, China; ^2^ School of Basic Medical Sciences, Heilongjiang University of Chinese Medicine, Haerbin, China; ^3^ Formula-Pattern Research Center, School of Traditional Chinese Medicine, Jinan University, Guangzhou, China

**Keywords:** depression, xiaoyaosan, NR2B, PI3K/Akt pathway, hippocampal CA1 region, glutamate

## Abstract

**Purpose:** It is revealed that Xiaoyaosan could reduce glutamate level in the hippocampus of depressed rats, whose metabolism leads to the pathophysiology of depression. However, the underlying mechanism remains unclear. This study aims to explore the effect of Xiaoyaosan on glutamate metabolism, and how to regulate the excitatory injury caused by glutamate.

**Methods:** Rats were induced by chronic unpredictable mild stress, then divided into control, vehicle (distilled water), Xiaoyaosan, fluoxetine, vehicle (DMSO), Xiaoyaosan + Ly294002 and Ly294002 groups. Ly294002 was microinjected into the lateral ventricular catheterization at 5 mM. Xiaoyaosan (2.224 g/kg) and fluoxetine (2.0 mg/kg) were orally administered for three weeks. The open field test (OFT), forced swimming test (FST), and sucrose preference test (SPT) were used to assess depressive behavior. The glutamate and corticosterone (CORT) levels were detected by ELISA. Western blot, immunochemistry or immunofluorescence were used to detect the expressions of NR2B, MAP2, PI3K and P-AKT/Akt in the hippocampal CA1 region. The mRNA level of MAP2, NR2B and PI3K were detected by RT-qPCR.

**Results:** Compared to the rats in control group, body weight and food intake of CUMS rats was decreased. CUMS rats also showed depression-like behavior as well as down regulate the NR2B and PI3K/Akt signaling pathway. Xiaoyaosan treatments could increase food intake and body weight as well as improved time spent in the central area, total distance traveled in the OFT. Xiaoyaosan could also decrease the immobility time as well as increase the sucrose preference in SPT. Moreover, xiaoyaosan decreased the level of glutamate in the hippocampal CA1 region and serum CORT in CUMS rats. Furthermore, xiaoyaosan improved the expression of MAP2 as well as increased the expression of NR2B, PI3K and the P-AKT/AKT ratio in the hippocampal CA1 region in the CUMS rats.

**Conclusion:** Xiaoyaosan treatment can exert the antidepressant effect by rescuing hippocampal neurons loss induced by the glutamate-mediated excitotoxicity in CUMS rats. The underlying pathway maybe through NR2B and PI3K/Akt signaling pathways. These results may suggest the potential of Xiaoyaosan in preventing the development of depression.

## Introduction

Depression is a common illness worldwide and is characterized by a decreased will, cognitive dysfunction and persistent mood depression. Major depression can lead to suicide. According to the latest statistics from the World Health Organization (WHO), nearly 800,000 depression patients die due to suicide annually [www.who.int]. However, current antidepressants have some limitations, such as low response rates and long treatment times, and some side effects (Rush et al., 2006; Trivedi et al., 2006).

Xiaoyaosan, which is a classic formula in traditional Chinese medicine (TCM), was first recorded in Taiping Huimin Heji Jufang during the Song dynasty. Xiaoyaosan has been used for the treatment of depression for more than 2000 years in China. It has been demonstrated that Xiaoyaosan can decrease the Hamilton Depression Rating Scale (HAMD) and Self-Rating Depression Scale (SDS) scores in depression patients (Feng et al., 2014; Feng et al., 2016). Xiaoyaosan has been demonstrated to ameliorate chronic unpredictable mild stress (CUMS)-induced depression-like behaviors (Liu et al., 2019) and promote neuronal plasticity in HT22 cells. Xiaoyaosan can protect against corticosterone-induced stress injury in primary hippocampal neurons (PHNs) (Cao et al., 2016). In addition, the glutamate content was increased in the hippocampus of rats with depression-like behavior (Dong et al., 2010), and Xiaoyaosan reportedly has the ability to decrease the level of glutamate (Liu et al., 2019).

Glutamic acid (Glu) mediates most central nerve excitatory conduction. The N-methyl-D-aspartate receptor (also known as the NMDA receptor or NMDAR) NMDA receptors constitute a class of ionic glutamate receptors. NMDA receptors family includes GluN1, GluN2 (NR2A, NR2B, NR2C, NR3D) and GluN3 (NR3BA and NR3B). However, NR2B was demonstrated to be the most strongly associated with depression (Duman and Voleti, 2012; Wang et al., 2014). In addition, the downregulation of NR2B expression can trigger multiple signaling pathways; among these pathways, phosphoinositide 3-kinase (PI3K) can be induced by the NMDA receptor (Waxman and Lynch, 2005; Kim et al., 2011; Wang et al., 2011a; Wang et al., 2011b). The PI3K/Akt pathway plays a role in depression. Studies have found that decreased Akt activity and decreased extracellular regulatory kinase activity coexist in depression, and autopsy studies have found that the activity of the PI3K-Akt signaling pathway is decreased in suicidal patients with major depression (Karege et al., 2011). PI3K and the P-AKT/AKT ratio were decreased in CUMS mice (Guo et al., 2019). Studies have demonstrated that isoflurane-induced neuroapoptosis could decrease the P-AKT/AKT ratio in the hippocampal CA1 region in neonatal rats (Li et al., 2014).

Depression has been shown to be a neurodegenerative disease. As a key brain region involved in memory and emotional information processing, the hippocampus is sensitive to stress and can be easily damaged. The hippocampus is among several marginal structures of depression that have been deeply studied. Of all brain regions, the hippocampus CA1 region is essential for cognition and spatial learning (Morris et al., 1982; Hayashi et al., 2015). The hippocampal CA1 region is extremely vulnerable and undergoes degeneration in patients with depression. Hippocampal CA1 damage occurring under pathological conditions contributes to neurological diseases (Bartsch et al., 2011). Therefore, this study focused on the hippocampal CA1 region in rats and attempted to identify an effective and safe approach for antidepression research. The use of traditional Chinese herbs has evolved over two thousand years in China and may fill this need because of their low risk of adverse events and potential combination therapy effects.

MAP2 is a neuron-specific cytoskeletal protein used as a marker of neuronal phenotypes (Izant and McIntosh, 1980). Previous studies have shown that Xiaoyaosan can increase the content of MAP2 in the hippocampus in 21-day-old chronic immobilization stress (CIS) rats (Liu, 2016). Whether Xiaoyaosan can promote the growth of neurons in the hippocampal CA1 region or increase the expression of MAP2 in CUMS rats needs further verification.

Although, our previous studies have shown that Xiaoyaosan could reduce the level of glutamate in the hippocampus of depressed rats, whose metabolism and reuptake into neurons in the brains leads the pathophysiology of depression, the underlying mechanism remains unclear. In the current study, we used MAP2 as a marker of neuronal phenotypes to detect the glutamate-induced toxicity in the hippocampal CA1 region of CUMS rats, and hypothesized that the Xiaoyaosan may alleviates hippocampal glutamate-induced toxicity in CUMS rats through NR2B and PI3K/Akt signaling pathway.

## Materials and Methods

### Animals

Healthy male Sprague-Dawley rats with body weights of 180–220 g (6–7 weeks old) were purchased from Beijing Vital River Laboratory Animal Research Center [animal license No. SCXK (Beijing) 2016-0006]. All animal experiments were approved by the Institutional Animal Care and Use Committee of Beijing University of Chinese Medicine and conformed to the guidelines for animal welfare (BUCM-4-2017051015-2015) to minimize animal suffering and animal use. The rats were maintained in the Specific Pathogen-Free (SPF) animal facility at the Beijing University of Chinese Medicine [certification number SYXK (Beijing) 2016-0038] (temperature: 20–22°C; relative humidity: 30–40%), given free access to distilled water and fed a regular rodent diet. The rats were allowed to habituate for one week before the experiments. Then, the rats were randomly divided into the following seven groups with 18 rats per group:

Control: No Stress.
Vehicle (W): CUMS + Distilled Water.
Xiaoyaosan: CUMS + Xiaoyaosan.
Fluoxetine: CUMS + Fluoxetine.
Vehicle (D): CUMS + DMSO.
Xiaoyaosan + Ly294002: CUMS + Ly294002 + Xiaoyaosan.
Ly294002 Groups: CUMS + Ly294002

### Surgical Procedures and Intracerebroventricular Microinjections

The rats in the vehicle (D), Xiaoyaosan + Ly294002 and Ly294002 groups were anaesthetized (1% sodium pentobarbital, 0.4 ml/100 g, i. p.), and then, lateral ventricular catheterization was performed. A microsyringe cannula was implanted in the lateral ventricle (AP: 1.0 mm; ML: 2.0 mm; DV: 3.5 mm) of the rats under a stereotactic apparatus. After the surgery, the rats were placed in an incubator to maintain their basal body temperature and then returned to their cage after they naturally awoke. Ketoprofen was administered to the rats (10 mg/kg/day for 3 days, i. m.) to relieve pain. The rats were allowed to recover for one week, and then, they were exposed to CUMS.

### CUMS Model Establishment

The rat model of stress was produced by exposing the rats to CUMS (Willner, 1997). CUMS was administered, excluding 24 h-duration stressors. The selected stress items were as follows: 1) 24-h water deprivation, 2) 24-h food deprivation, 3) 5-min heat at 45°C, 4) 24-h moist padding, 5) 17-h cage tilt at 45° from the horizontal, 6) 17-h white noise, 7) overnight illumination, 8) 1-min tail pinch, 9) 3-h physical restraint and 10) 17-h strange object exposure. The stressors were randomly assigned over 6 weeks to ensure that the same type of stressor did not occur on two consecutive days to prevent the animals from anticipating the occurrence of the stimuli.

### Preparation of Xiaoyaosan

Xiaoyaosan comprises the following eight herbs: Radix *Paeoniae alba* (root of *Paeonia lactiflora* Pall.), Radix *Bupleuri* (root of *Bupleurum chinense* DC. or *Bupleurum scorzonerifolium* Willd.), Rhizoma *Atractylodis macrocephalae* (rhizome of *Atractylodes macrocephala* Koidz.), Radix *Angelicae sinensis* (root of *Angelica sinensis* (Oliv.) Diels), Rhizoma Zingiberis (rhizome of *Zingiber officinale* Rosc.), Poria (dry sclerotia of *Pori cocos* (Schw.) Wolf.), Radix Glycyrrhizae (root of *Glycyrrhiza uralensis* Fisch.), and Herba *Menthae haplocal* (Herba *of Mentha haplocalyx* Briq.) at a ratio of 5:5:5:5:5:5:4:1. Xiaoyaosan (batch number J2447) was provided by Jiuzhitang Co., Ltd. (Changsha, China) and was prepared as previously described (Yuan et al., 2020). One gram of fine powder of Xiaoyaosan was obtained from 2.10 g of raw herbs. The phytoconstituents of Xiaoyaosan were detected by UPLC-MS (Dionex Utimate 3000 UHPLC Plus Focused coupled to an LTQ/Orbitrap MS system, Thermo Scientific, United States).

### Drug Administration

**FIGURE 1:**
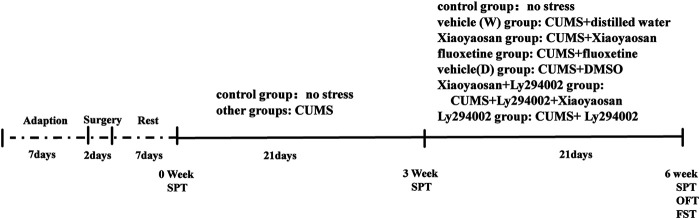
Timeline of the experiment. Before the experiment, the animals were allowed a 7-days adaptation period. The rats in the surgery groups underwent lateral ventricular catheterization and were allowed to recover for one week. Except for the control group, the rats in the other groups were subjected to CUMS for 6 weeks and treated with different drugs from the fourth week to the sixth week. The body weights and food intake levels were monitored weekly. A SPT was performed on day 0 (baseline SP test), day 21 and day 41. The OFT and FST were performed in week 6. Then, the rats were sacrificed for further detection.

The drug administration was performed after three weeks of the CUMS modeling (from the fourth week to the sixth week). CUMS was continued simultaneously with the drug intervention. The rats in the Xiaoyaosan and Xiaoyaosan + Ly294002 groups were treated with Xiaoyaosan daily (2.224 g/kg/d, 10 ml/kg body weight) by gavage. The rats in the fluoxetine group were treated with fluoxetine (2 mg/kg/d, 10 ml/kg body weight, Patheon France, packaged by Lilly Suzhou Pharmaceutical Co., Ltd., Suzhou, China), and the rats in the vehicle (W) group received distilled water (10 ml/kg body weight) by gavage (Hou et al., 2020). The rats in the Ly294002 and Xiaoyaosan + Ly294002 groups received an intracerebroventricular microinjection of Ly294002 (5 mM dissolved in DMSO) daily at a volume of 10 μL at a speed of 1 μL/min (Yu et al., 2012). The rats in the vehicle (D) group received an equal volume of DMSO daily by intracerebroventricular microinjections. The detailed timeline is shown in Figure 1.

## Behavioral Testing

### Open Field Test

The OFT was conducted as previously described (Ma et al., 2019). An open field box (100 cm × 100 cm × 50 cm) with a black floor and four black sidewalls was placed in the middle of a dark and quiet operation room, and a video camera connected to a computer was placed 1.5 m above the middle of the box. The rats were placed in the testing room for 30 min before the experiment. Each rat was gently placed in the center of the testing apparatus and allowed to freely explore for 5 min. After each test, the bottom of the apparatus was cleaned with low-concentration ethanol. The activity of the rats, including the total movement distance and time spent in the central area, was tracked and measured by software (supermase, Animal behavior video analysis software, Shanghai Xin Soft Information Technology Co., ltd.).

### Sucrose Preference Test

The SPT was carried out on day 0 (as baseline), day 21 and day 41 of the experimental process according to previously described methods (Ma et al., 2019). Briefly, the rats were deprived of food and water before the SPT. One bottle of distilled water and one bottle of 1% sucrose solution were weighed and marked in advance. Two identical drinking bottles were given to the rats for 1 h. Then, the bottles were simultaneously removed and measured. The sucrose preference was calculated by the following formula: sucrose preference = sucrose consumption/[total consumption (sucrose + water)] × 100% (Xu et al., 2020).

### Forced Swimming Test

The FST was carried out as previously described (Liu et al., 2019). The rats were gently and slowly placed in cylindrical water tanks (80 cm height × 40 cm diameter, temperature of water 23 ± 1°C). The rats were allowed to stay in the tanks for 6 min. The immobility time during the last 5 min was recorded by experimenters who were blinded to the design.

### Tissue Sample Collection

After 6 weeks, the whole brains of 42 rats (6 rats randomly selected from each group) were fixed with 4% paraformaldehyde via arterial perfusion under deep anaesthesia (1% sodium pentobarbital 0.4 ml/100 g, i. p.) and stored in 4% paraformaldehyde solution at four degree centigrade for paraffin embedding. The remaining rats were sacrificed by decapitation under anaesthesia with 1% sodium pentobarbital (0.4 ml/100 g, i. p.). The whole brains of the remaining rats obtained without perfusion were sectioned into 1.0 mm consistently sliced coronal sections using an Adult Rat Brain Slicer Matrix (BSRAS001-1, ZIVIC Instrument, United States). Under an anatomical microscope (SMZ-171-BLED Stereo, Motic, United States), hippocampal-CA1 region tissues were removed, collected in frozen pipes and stored at −80°C for the western blot, RT-qPCR and ELISA analyses.

### ELISA

The level of glutamate in the hippocampal CA1 region was determined by ELISA following the manufacturer’s instructions (Bio- Assay Systems, #EGLT-100). The level of corticosterone (CORT) in the serum was determined by ELISA following the manufacturer’s instructions (Enzo ADI-900-097).

### Quantitative Real-Time Polymerase Chain Reaction

The mRNA levels of NR2B, PI3K, and MAP2 were detected by RT-qPCR and the primers were designed according to Primer-premier five and BLAST. The total hippocampal RNA was extracted by an RNA extraction kit, followed by cDNA synthesis with a RevertAid First Strand cDNA Synthesis Kit (Thermo Scientific, United States) using a Mastercycler Gradient thermal cycler (Eppendorf, Germany). The primer sequences shown in Table 1 were synthesized by Sangon Biotech Co., Ltd. (Shanghai, China). RT-qPCR was performed by using Power SYBR® Green PCR Master Mix (Thermo Fisher Scientific, United States) at a 20 ml volume using a CFX96™ Real-Time System (Bio-Rad, United States) with the following amplification cycling protocol: 95°C for 10 min, 40 cycles of 95°C for 15 s, and 60°C for 60 s. The relative quantitative analysis of the RT-qPCR results was performed by Bio-Rad CFX Manager 2.1 (Bio-Rad, United States). The mRNA levels were normalized to GAPDH and calculated using the 2^−△△Ct^ method.

**TABLE 1:** Primer sequences.

Gene	—	Sequences
PI3K	Forward	AAA​GTT​TCA​GGC​AGC​AGT​GG
	Reverse	TCG​TTG​TGC​CTG​TCA​CCT​AT
NR2B	Forward	TGC​TCA​ACT​ACA​TGG​CTG​GA
	Reverse	AAG​CAA​AGA​CCT​TGC​CAC​TG
MAP2	Forward	AGT​TGC​CAT​CAT​TCG​CAC​TC
	Reverse	TTC​TTC​AGG​TCT​GGC​AGA​GG
GAPDH	Forward	CCA​TTC​TTC​CAC​CTT​TGA​T
	Reverse	TGG​TCC​AGG​GTT​TCT​TAC​T

### Western Blot Analysis

Western blot analysis was performed as previously described (Liu et al., 2018). The hippocampal CA1 areas of the rats were homogenized in RIPA buffer (with a protease inhibitor) (Beyotime Biotechnology, Shanghai, China). The protein concentration was determined by a BCA protein assay kit (Thermo Scientific, United States). The samples (50 or 100 μg) were run on a 10% SDS-PAGE gel and transferred onto PVDF membranes. The PVDF membranes were blocked with 5% skim milk in TBST (Tris-buffered saline containing 0.1% Tween 20) for 1 h at room temperature and incubated overnight at 4°C with the primary antibodies [PI3K (Proteintech, 60225-1-Ig, 1:3,000), Akt (Abcam, ab238477 1:1,000), P-AKT (Abcam, ab81283, 1:4,000), MAP2 (Abcam, ab5392,1:5,000) and NR2B (Affinity AF6426, 1:1,000)]. The PVDF membranes were washed three times with TBST, followed by incubation with an HRP-labelled secondary antibody (1:5,000) at room temperature for 2 h. The membranes were washed three times with TBST and visualized with high sensitivity ECL luminous liquid before imaging using an Azure Bioimaging system (California, USA). The results were quantified by ImageJ software. β-actin (1:5,000) was used as an internal control.

### Immunohistochemical Staining and Immunofluorescence

IHC staining was conducted as previously described (Li et al., 2019) with some modifications. Five-micrometre paraffin-embedded brain tissue samples were placed in an oven at 60°C for 4 h, followed by defatting with xylene and dehydrating with graded ethanol (100–50%). Then, the slides were boiled in water at 100°C for 10 min, followed by washing with TBST three times. Then, the slides were blocked with 3% hydrogen peroxide for 20 min at room temperature (RT). After washing with TBST three times, the sections were incubated with a primary antibody for 16 h [NR2B (1:50), PI3K (1:200) or P-Akt (1:300)]. After washing with TBST three times, the sections were incubated with a secondary antibody (Beijing Biosynthesis Biotechnology Co. Ltd., China) for 30 min at 37°C, followed by DAB and hematoxylin staining. The slides used for the IF staining were incubated with primary antibodies against MAP2 (Abcam, ab5392,1:1,500) at 4°C. After 18 h, all slides were incubated with fluorescent-conjugated goat anti-chicken IgG, followed by DAPI staining. The sections were photographed under an Olympus BX53 fluorescence microscope and analyzed by Image-Pro Plus 6.0 software (Rockville, Unites States).

### Statistical Analysis

All data obtained from the animal experiments using rats in different groups are expressed as the mean ± standard deviation (SD). One-way analysis of variance (ANOVA) was used when the data exhibited a normal distribution, and the variances were homogeneous. The body weight and food intake data were analyzed using repeated-measures ANOVA to determine significant differences considering time and stress (version 20.0, IBM SPSS Statistics). Statistical significance was determined as a *p*-value < 0.05.

## Results

### Characterization of the Main Phytoconstituents of Xiaoyaosan

Several phytoconstituents of Xiaoyaosan were identified by UPLC-MS, and as shown in Supplement Figures 1A,B, the following seven compounds were identified: 1) paeoniflorin, 2) liquiritin, 3) isoliquiritin, 4) liquiritigenin, 5) saikosaponin A, 6) curcumin and 7) ferulic acid.

### Xiaoyaosan Increased the Body Weight and Food Intake of CUMS Rats

**FIGURE 2:**
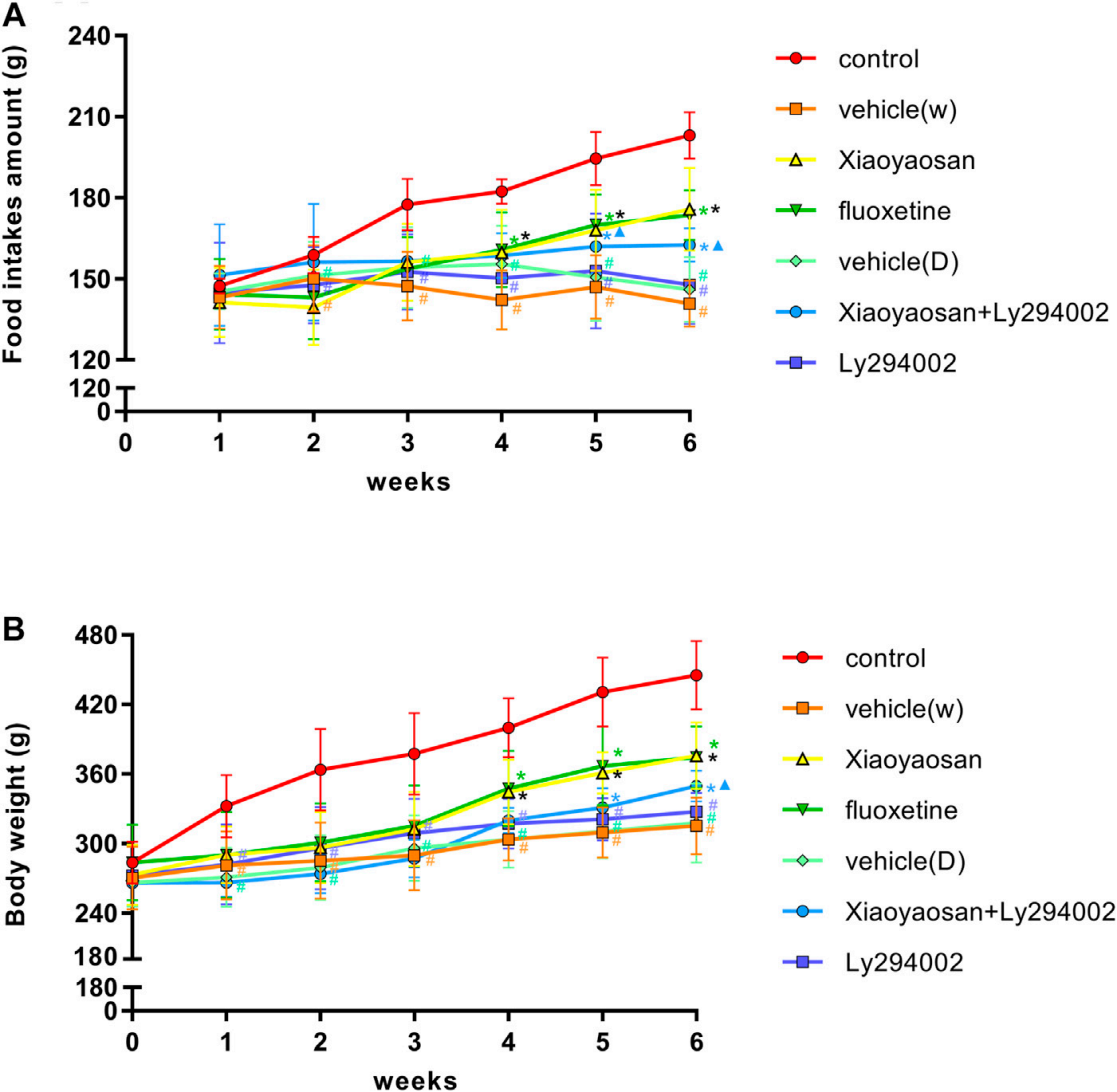
Changes in the body weight and food intake of the CUMS rats. **(A)** Changes in food intake from week one1 to week 6. **(B)** Changes in body weight from week 0 to week 6. The data are expressed as the mean ± SD. #*p* < 0.05 compared to the control group; *p < 0.05 compared to the vehicle (W) group; ▲*p* < 0.05 compared to the Ly294002 group, *n* = =18.

The rats’ body weight and food intake were affected throughout and after the stress period. As shown in Figure 2A, the food intake levels during the second week to the sixth week of the rats in the vehicle (W), vehicle (D) and Ly294002 groups were lower than those of the rats in the control group (*p* < 0.05). Specifically, the food intake of the rats decreased from 150.1 ± 12.2 g, 151.1 ± 12.6 g and 147.7 ± 14.2 g in the second week to 140.8 ± 8.4 g, 146.0 ± 12.0 g and 147.8 ± 14.5 g at the end of experiment in the vehicle (W), vehicle (D) and Ly294002 groups, respectively. The food intake of the rats increased from 158.7 ± 6.8 g in the second week to 194.4 ± 9.9 g in the sixth week in the control group. Upon treatment with Xiaoyaosan (175.7 ± 15.3 g), fluoxetine (173.6 ± 9.2 g) and Xiaoyaosan + Ly294002 (162.5 ± 6.2 g), the food intake of the CUMS rats increased compared to that in the model group (140.8 ± 8.4 g, *p* < 0.05). The food intake of the rats in the Xiaoyaosan + Ly294002 group in week 5 (161.9 ± 10.1 g) and week 6 (162.5 ± 6.2 g) was higher than that in the Ly294002 group (fifth week 152.9 ± 21.2 g and sixth week 147.8 ± 14.5 g). There were no significant differences between the vehicle (W) and vehicle (D) groups (*p* > 0.05). Figure 2B shows that there was no significant difference in body weight among the seven groups of rats at the beginning. The body weights of the rats in the vehicle (W), vehicle (D) and Ly294002 groups were significantly lower than those of the rats in the control group (*p* < 0.05) from week one to week 6. Specifically, the body weight of the rats increased from 281.0 ± 29.3 g, 270.7 ± 25.5 g and 281.7 ± 34.4 g in the first week to 315.2 ± 24.5 g, 317.7 ± 34.2 g and 327.2 ± 16.3 g at the end of the experiment in the vehicle (W), vehicle (D) and Ly294002 groups, respectively. The body weight of the rats increased from 332.0 ± 27.0 g in the first week to 445.1 ± 29.6 g in the sixth week in the control group. Upon the administration of Xiaoyaosan (375.7 ± 28.8 g), fluoxetine (374.5 ± 26.6 g) and Xiaoyaosan + Ly294002 (349.5 ± 13.3 g), the body weights of the CUMS rats increased compared to those of the rats in the vehicle (W) group (315.2 ± 24.5 g, *p* < 0.05) at the end of the experiment. In week 6, the body weight of the rats in the Xiaoyaosan + Ly294002 group (349.5 ± 13.3 g) was higher than that of the rats in the Ly294002 group (327.2 ± 16.3 g, *p* < 0.05). There were no significant differences between the vehicle (W) group and the vehicle (D) group (*p* > 0.05).

### Xiaoyaosan Ameliorates CUMS Depression-like Behaviors in Rats

**FIGURE 3:**
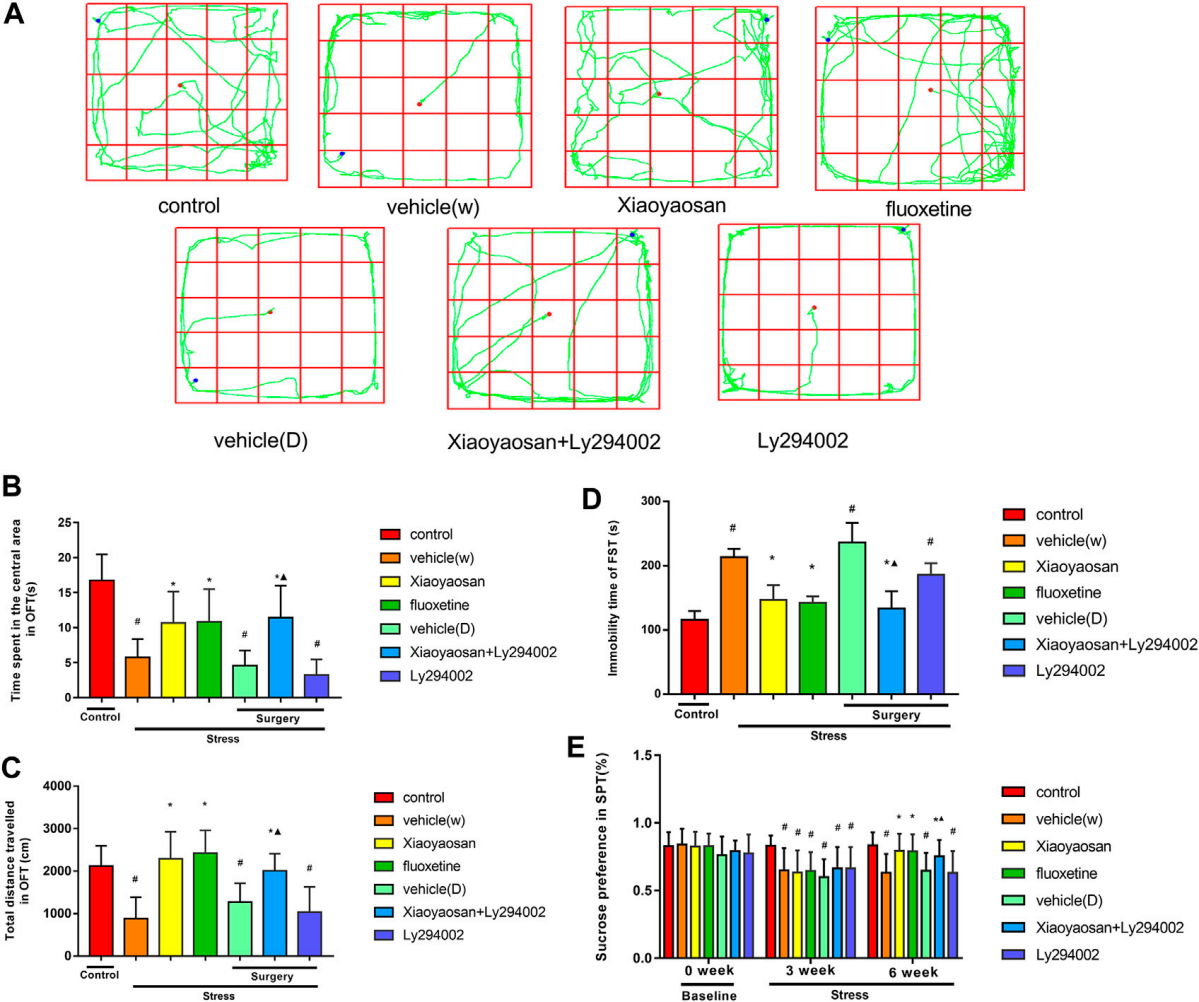
Xiaoyaosan ameliorates CUMS rat depression-like behaviors **(A)** Movement trajectory of each group of rats **(B)** Time spent in the central area in the OFT by the rats in each group **(C)** Total distance traveled within 5 min in the OFT by the rats in each group **(D)** Immobility time in the FST of the rats in each group **(E)** Sucrose preference in the SPT of the rats in each group in weeks 0, three and 6. All data are expressed as the mean ± SD. #*p* < 0.05 compared to the control group; *p < 0.05 compared to the vehicle (W) group; ▲*p* < 0.05 compared to the Ly294002 group, *n* = 18.

An OFT, FST and SPT were performed to evaluate the depression-like behaviors of the CUMS rats. The OFT is commonly used to reflect rodents’ exploration characteristics and fear of a new environment (Belovicova et al., 2017). In the OFT test (Figures 3A–C), the central region residence time of the rats in the vehicle (W), vehicle (D) and Ly294002 groups was shorter than that of the rats in the control group (*p* < 0.05). Specifically, the central region residence time of the rats in the vehicle (W), vehicle (D), Ly294002 and control groups were 5.8 ± 2.6 s, 4.6 ± 2.2 s, 3.2 ± 2.3 s and 16.7 ± 3.7 s, respectively. The rats in the Xiaoyaosan (10.7 ± 4.5 s), fluoxetine (10.8 ± 4.7 s) and Xiaoyaosan + Ly294002 (11.4 ± 4.6 s) groups spent more time in the central area than the rats in the vehicle (W) group (5.8 ± 2.6 s, *p* < 0.05). The rats in the Xiaoyaosan + Ly294002 group showed a longer central region residence than those in the Ly294002 group (*p* < 0.05). The total distances traveled by the rats in the vehicle (W), vehicle (D) and Ly294002 groups were shorter than that of the rats in the control group (*p* < 0.05). Specifically, the total distances traveled by the rats in the vehicle (W), vehicle (D), Ly294002 and control groups were 881.4 ± 504.1 cm, 1266.8 ± 443.3 cm, 1036.6 ± 593.4 cm and 2119.5 ± 480.4 cm, respectively. The rats in the Xiaoyaosan (2290.7 ± 634.4 cm), fluoxetine (2419.5 ± 538.4 cm) and Xiaoyaosan + Ly294002 (2008.1 ± 398.9) groups spent more time in the central area than those in the vehicle (W) group (881.4 ± 504.1 cm, *p* < 0.05). The total distance traveled in the OFT by the rats in the Xiaoyaosan + Ly294002 group was higher than that of the rats in the Ly294002 group (*p* < 0.05). There was no statistical difference in the OFT between rats in the Xiaoyaosan and Xiaoyaosan + Ly294002 groups in OFT. As shown in Figure 3D, the immobility times of the rats in the vehicle (W) (213.7 ± 12.9 s), vehicle (D) (236.7 ± 29.9 s) and Ly294002 groups (186.2 ± 17.9 s) were longer than those of the rats in the control group (115.8 ± 13.8 s, *p* < 0.05). The rats in the Xiaoyaosan (147.0 ± 23.2 s), fluoxetine (142.2 ± 10.1 s) and Xiaoyaosan + Ly294002 groups (133.7 ± 26.9 s) had a lower immobility time than the rats in the vehicle (W) group (213.7 ± 12.9 s, *p* < 0.05). The immobility time in the FST of the rats in the Xiaoyaosan + Ly294002 group was lower than that of the rats in the Ly294002 group (*p* < 0.05). The SPT is the gold standard for evaluating the degree of pleasure loss in rodents. Anhedonia is manifested by the lack of interest in a reward stimulus, which is a manifestation of affective disorders, including depression (Weiss, 1997; Belovicova et al., 2017). However, there was no statistical difference in the immobility time in the FST between rats in the Xiaoyaosan and Xiaoyaosan + Ly294002 groups. The SPT was performed on days 0, 21 and 41 (Figure 3E). The baseline sucrose preferences rates of the rats in the seven groups did not significantly differ. However, after 3 weeks, the CUMS rats in the vehicle (W) (64.8 ± 16.6%), Xiaoyaosan (63.2 ± 16.4%), fluoxetine (64.4 ± 14.0%), vehicle (D) (59.9 ± 13.2%), Xiaoyaosan + Ly294002 (66.2 ± 15.8%) and Ly294002 groups (66.2 ± 15.8%) exhibited a significant decrease in the sucrose preference rate compared to that of the rats in the control group (83.1 ± 7.6%, *p* < 0.05). The rats in the vehicle (W), vehicle (D) and Ly294002 groups had lower sucrose preference rates than the rats in the control group (*p* < 0.05). The treatment with Xiaoyaosan, fluoxetine and Xiaoyaosan + Ly294002 for 3 weeks reversed this trend, and the sucrose preference rates were increased compared to those of the vehicle (W) group (*p* < 0.05). Specifically, the sucrose preference rates in the Xiaoyaosan, fluoxetine, Xiaoyaosan + Ly294002 and vehicle (W) groups were 79.3 ± 12.6%, 78.9 ± 12.5%, 75.3 ± 12.0% and 63.0 ± 14.0%, respectively. The sucrose preference rate of the rats in the Xiaoyaosan + Ly294002 group was higher than that of the rats in the Ly294002 group (*p* < 0.05). There was no statistical difference in the sucrose preference rates between rats in the Xiaoyaosan and Xiaoyaosan + Ly294002 groups.

### Xiaoyaosan Decreased the Glutamate Level in the Hippocampal CA1 Area of the CUMS Rats

**FIGURE 4:**
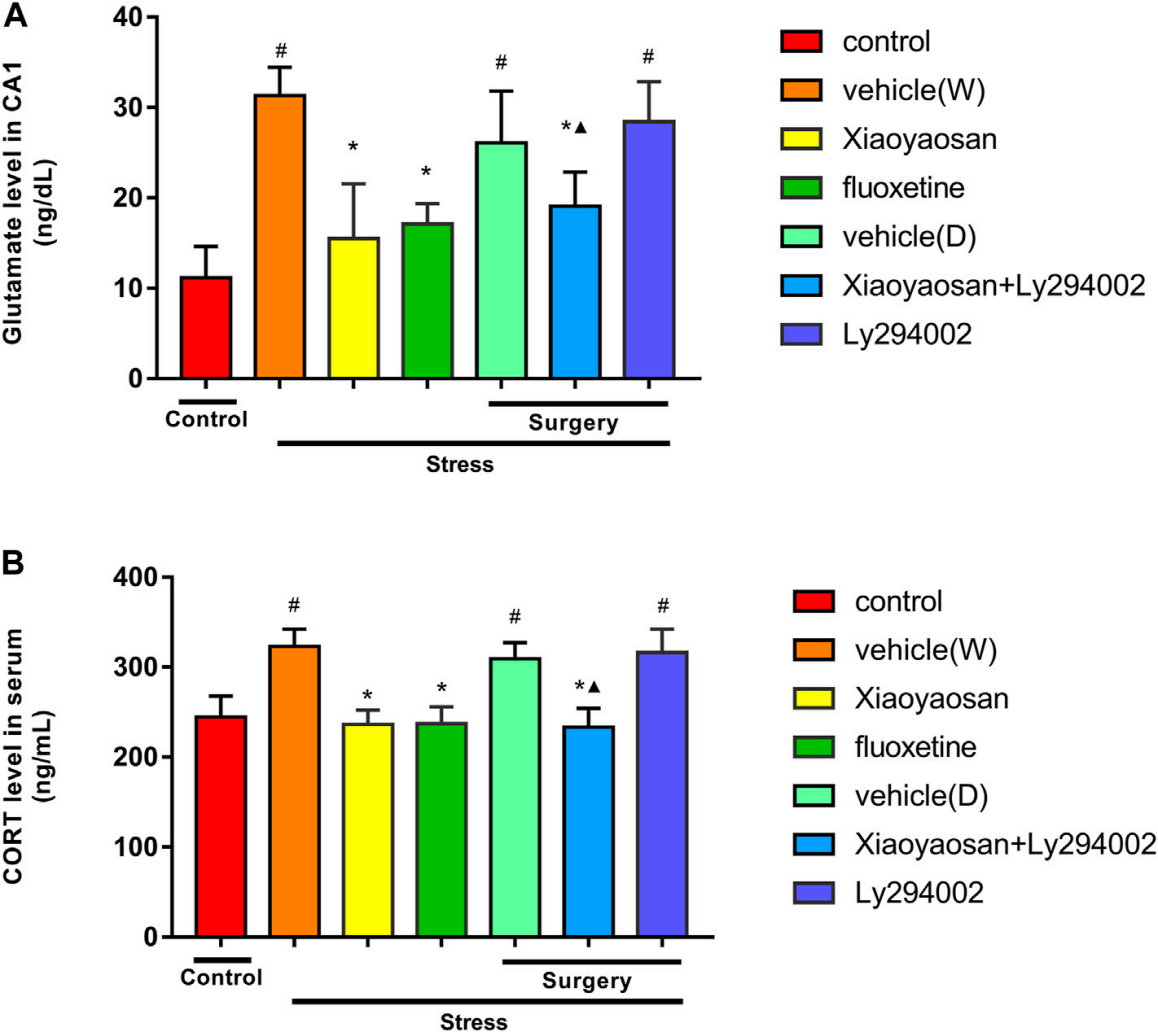
Levels of glutamate in the hippocampal CA1 area **(A)** and CORT in the serum **(B)** in the treatment and control groups of rats. The data are expressed as the mean ± SD. #*p <* 0.05 compared to the control group; *p < 0.05 compared to the vehicle (W) group; ▲*p* < 0.05 compared to the Ly294002 group, *n* = 6.

As shown in Figure 4A, the level of glutamate in the hippocampal CA1 area of the rats exposed to CUMS was significantly higher than that that of the rats in the control group (*p* < 0.05). The Xiaoyaosan, fluoxetine and Xiaoyaosan + Ly294002 treatments significantly reversed these alterations by decreasing the levels of glutamate by 50.6, 45.3 and 39.2%, respectively, compared to the levels in the vehicle (W) group (*p* < 0.05). Compared to those in the control group, the rats in the vehicle (D) and Ly294002 groups exhibited increased levels of glutamate in the hippocampal CA1 area by 134.2 and 155.3%, respectively (*p* < 0.05). The level of glutamate in the hippocampal CA1 area of the rats in the Xiaoyaosan + Ly294002 group was lower than that in the Ly294002 group (*p* < 0.05).

### Xiaoyaosan Decreased the CORT Level in Serum of the CUMS Rats

Corticosterone (CORT), a glucocorticoid of the adrenal cortical hormones secreted by the adrenal glands in rats, which disordered secretion was associated with neurodegenerative injury and regeneration disorder (Wu et al., 2017; Nandam et al., 2019). Our previous study also demonstrated that chronic stress could cause HPA axis negative feedback disorder (Li, 2018). In the present study, we detected the serum CORT level by ELISA. As shown in Figure 4B, the level of CORT in serum of the rats exposed to CUMS was significantly higher than those of in the control group (*p* < 0.05). The Xiaoyaosan, fluoxetine and Xiaoyaosan + Ly294002 treatments significantly reversed these alterations by decreasing the levels of CORT, compared to the levels in the vehicle (W) group (*p* < 0.05). Compared to those in the control group, the rats in the vehicle (D) and Ly294002 groups increased CORT level in serum of rats (*p* < 0.05). The level of CORT in serum of the rats in the Xiaoyaosan + Ly294002 group was lower than that in the Ly294002 group (*p* < 0.05).

### Xiaoyaosan Increased the Expression of MAP2 in the Hippocampal CA1 Region of the CUMS Rats

**FIGURE 5:**
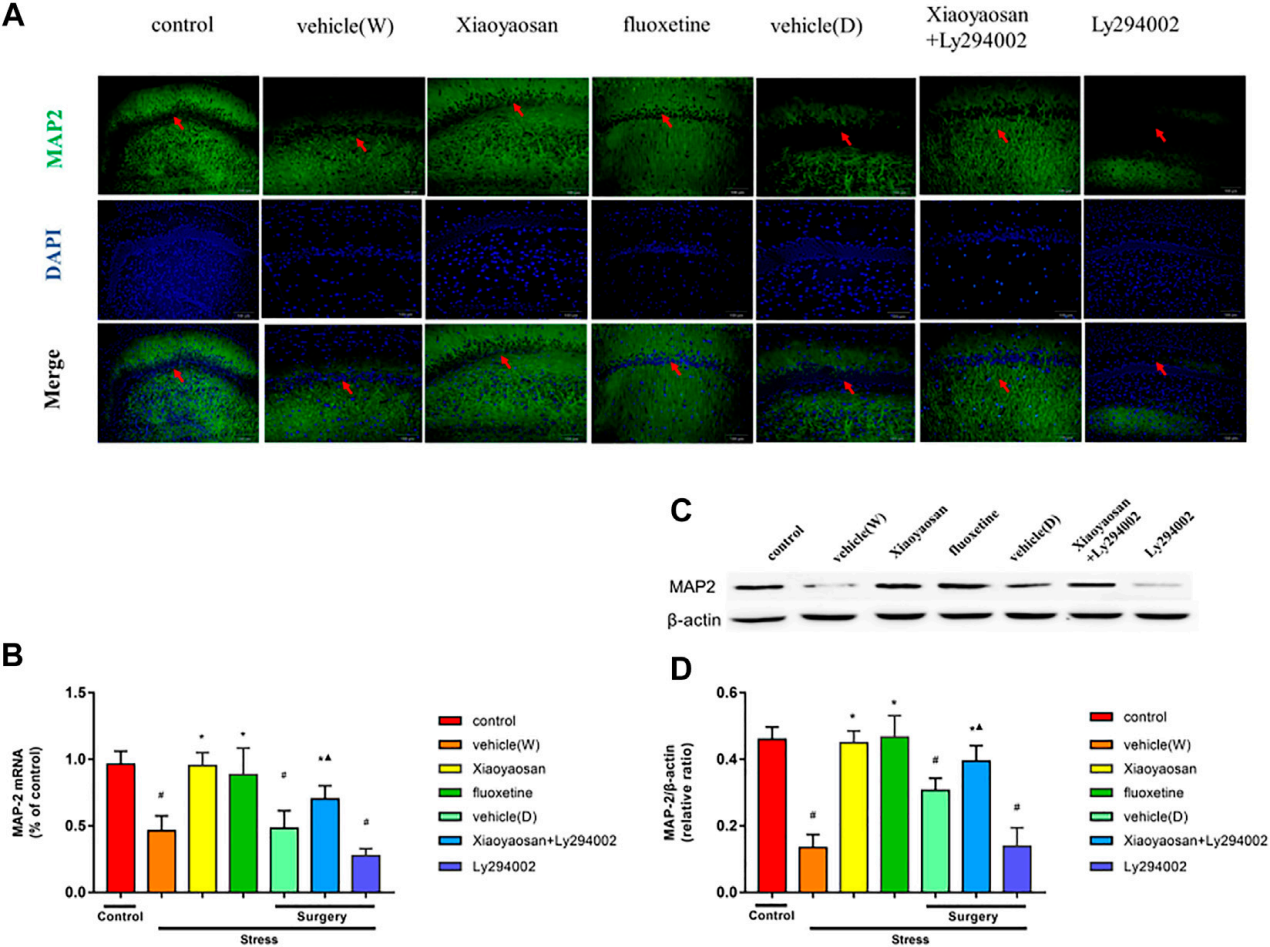
Xiaoyaosan elevates the expression of MAP2 in the hippocampal CA1 region of CUMS rats (**A**, original magnifification, ×200) Xiaoyaosan and Xiaoyaosan + Ly294002 promoted MAP2 expression in the CUMS rats. The green color represents MAP2 staining, and the blue color represents nuclear staining **(C)** Representative images and western blot analysis **(D)** of western blot assay showing the relative expression of MAP2 **(B)** Level of MAP2 mRNA in the hippocampal CA1 area of the rats in the treatment and control groups. All data are expressed as the mean ± SD. ^#^ *p* < 0.05 compared to the control group; ^*^ *p* < 0.05 compared to the vehicle (W) group; ^▲^ *p* < 0.05 compared to the Ly294002 group, *n* = 6.

MAP2 is a neuron-specific marker (Dinsmore and Solomon, 1991; Caceres et al., 1992; Sharma et al., 1994). In this study, we investigated the changes of MAP2 expression in the hippocampal CA1 region in the CUMS rats. As shown in Figure 5A, MAP2 labeled with green fluorescence was distributed in the hippocampal CA1 area in the CUMS rats. The nuclei were stained blue with DAPI. The expression of MAP2 in the rat hippocampal CA1 area in the vehicle (W), vehicle (D) and Ly294002 groups was lower than that in the control group. However, upon the treatment with Xiaoyaosan, fluoxetine or Xiaoyaosan + Ly294002 for 3 weeks, the MAP2 expression in the CA1 region of the hippocampus in the CUMS rats significantly increased. The RT-qPCR (Figure 5B) results showed that the expression of MAP2 in the hippocampal CA1 region of the rats in the vehicle (W), vehicle (D) and Ly294002 groups was lower than that of the rats in the control group (*p* < 0.05). Upon the treatments with Xiaoyaosan, fluoxetine and Xiaoyaosan + Ly294002, the level of MAP2 mRNA in the hippocampal CA1 region of the rats was higher than that of the rats in the vehicle (W) group (*p* < 0.05). The expression of MAP2 in the hippocampal CA1 region of the rats in the Xiaoyaosan + Ly294002 group was lower than that of the rats in the Ly294002 group (*p* < 0.05). The western blot (Figures 5C,D) results were consistent with the immunofluorescence and RT-qPCR results.

### Xiaoyaosan Increased the Expression of NR2B in the Hippocampal CA1 Area of the CUMS Rats

**FIGURE 6:**
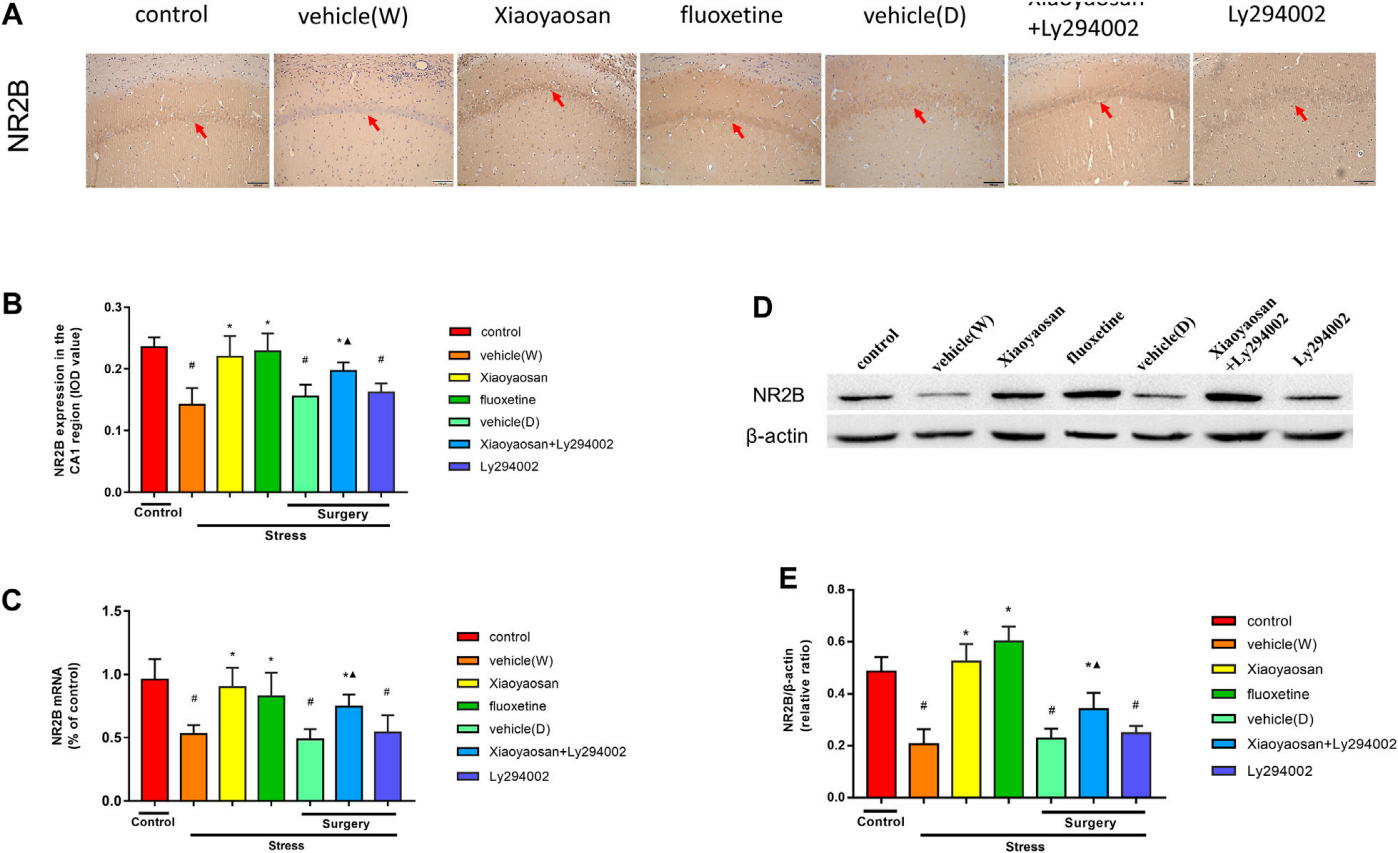
Xiaoyaosan elevates the expression of NR2B in the hippocampal CA1 region of CUMS rats **(A)** Representative micrographs of immunohistochemical staining (sections were counterstained with hematoxylin; original magnification, ×200) and **(B)** quantitative analysis showing the expression of NR2B in the hippocampal CA1 region **(C)** NR2B mRNA level in the hippocampal CA1 region **(D)** Representative images and western blot analysis **(E)** of western blot assay showing the relative expression of NR2B in the hippocampal CA1 region. All data are expressed as the mean ± SD. #*p* < 0.05 compared to the control group; *p < 0.05, compared to the vehicle (W) group; ▲*p* < 0.05 compared to the Ly294002 group, *n* = 6.

As shown in Figure 6C, we examined the level of NR2B mRNA in the hippocampal CA1 region of the rats by RT-qPCR. The level of NR2B in the rat hippocampal CA1 area in the vehicle (W), vehicle (D) and Ly294002 groups was lower than that that in the control group (*p* < 0.05). However, upon the treatment with Xiaoyaosan, fluoxetine or Xiaoyaosan + Ly294002 for 3 weeks, the level of NR2B mRNA in the CA1 region of the hippocampus in the CUMS rats significantly increased (*p* < 0.05). Compared to the rats in the Ly294002 group, the rats in the Xiaoyaosan + Ly294002 treatment exhibited a reverse of the decrease in the level of NR2B mRNA in the CA1 region of the hippocampus of the rats (*p* < 0.05). There was no difference between the rats in the vehicle (W) and vehicle (D) groups. As shown in Figures 6A,B, the expression of NR2B in the rat hippocampal CA1 area in the vehicle (W), vehicle (D) and Ly294002 groups was lower than that of the rats in the control group (*p* < 0.05). Interestingly, upon the treatment with Xiaoyaosan, fluoxetine or Xiaoyaosan + Ly294002 for 3 weeks, the expression of NR2B in the CA1 region of the hippocampus in the CUMS rats significantly increased (*p* < 0.05). Compared to the rats in the Ly294002 group, the rats in the Xiaoyaosan + Ly294002 group exhibited a reverse of the decrease in the expression of NR2B in the CA1 region of the hippocampus of the rats (*p* < 0.05). The western blot results (Figures 6D,E) were consistent with the immunohistochemistry results.

### Effect of Xiaoyaosan on the PI3K/AKT Pathway in the Hippocampal CA1 Region of the CUMS Rats

**FIGURE 7:**
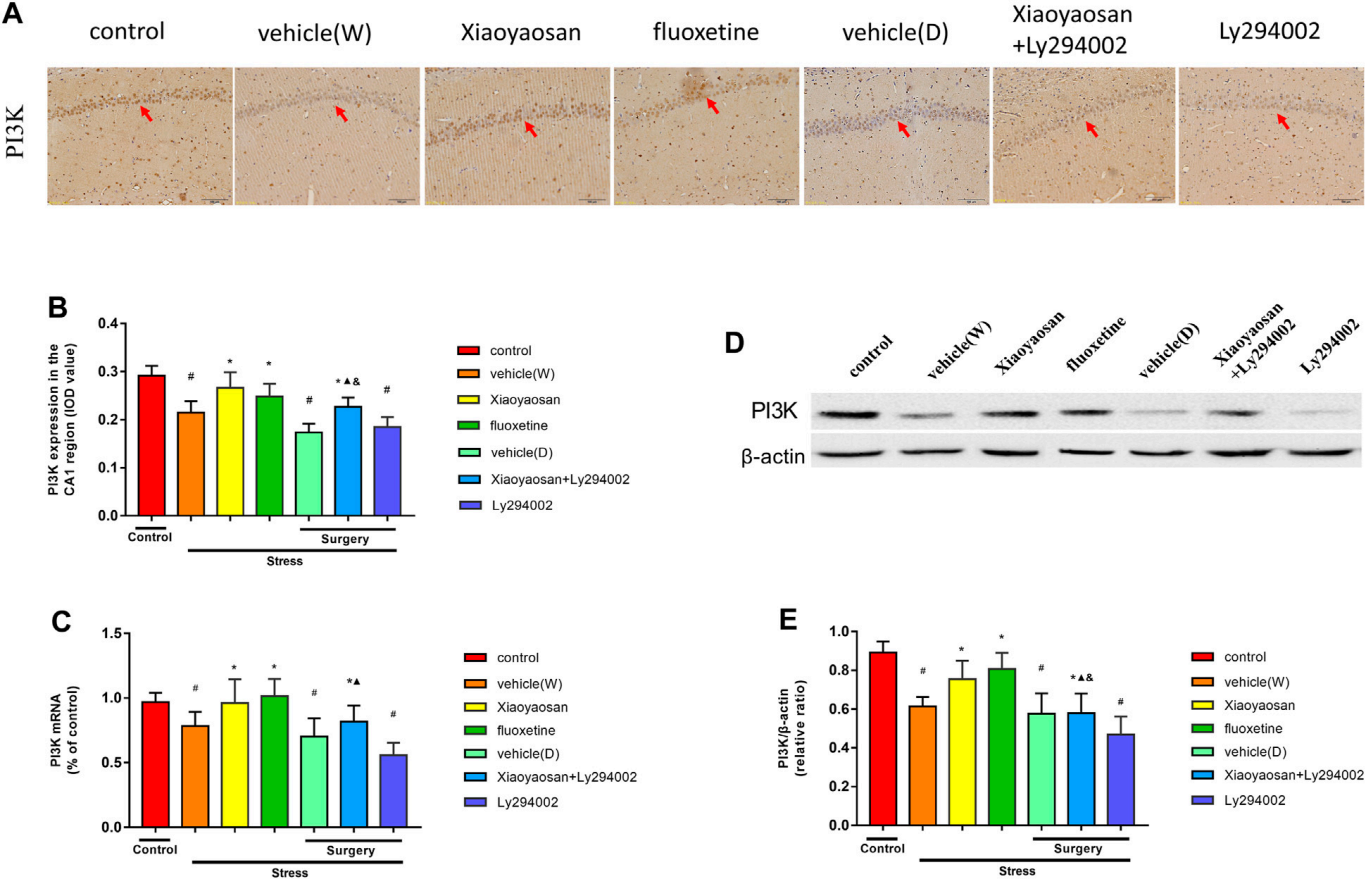
Xiaoyaosan elevates the expression of PI3K in the hippocampal CA1 region of CUMS rats **(A)** Representative micrographs of immunohistochemical staining (sections were counterstained with hematoxylin; original magnification, ×200) and **(B)** quantitative analysis showing the expression of PI3K in the hippocampal CA1 region **(C)** Level of PI3K mRNA in the hippocampal CA1 region **(D)** Representative images and western blot analysis **(E)** of western blot assay showing the relative expression of PI3K in the hippocampal CA1 region. All data are expressed as the mean ± SD. #*p* < 0.05 compared to the control group; *p < 0.05, compared to the vehicle (W) group; ▲*p* < 0.05 compared to the Ly294002 group; ^&^ *p* < 0.05 compared to the Xiaoyaosan group, *n* = 6.

To further explore the potential mechanism of Xiaoyaosan in CUMS rats, PI3K was observed in the rat hippocampal CA1 area by RT-qPCR, western blot and IHC staining analyses. As shown in Figures 7A,B, the expression of PI3K in the rat hippocampal CA1 area in the vehicle (W), vehicle (D) and Ly294002 groups was lower than that in the control group (*p* < 0.05). Interestingly, upon treatment with Xiaoyaosan, fluoxetine or Xiaoyaosan + Ly294002 for 3 weeks, the expression of PI3K in the CA1 region of the hippocampus in the CUMS rats significantly increased (*p* < 0.05). Compared to that of the rats in the Ly294002 group, the expression of PI3K in the CA1 region of the hippocampus of the rats in the Xiaoyaosan + Ly294002 group was higher (*p* < 0.05). Compared to the rats in the Xiaoyaosan group, the expression of PI3K in the CA1 region of the hippocampus of the rats in the Xiaoyaosan + Ly294002 group was lower (*p* < 0.05). Figure 7C showed the level of the PI3K gene in the hippocampal CA1 region of the rats by RT-qPCR. The level of PI3K in the rat hippocampal CA1 area in the vehicle (W), vehicle (D) and Ly294002 groups was lower than that of the rats in the control group (*p* < 0.05). However, upon the treatment with Xiaoyaosan, fluoxetine or Xiaoyaosan + Ly294002 for 3 weeks, the level of PI3K mRNA in the CA1 region of the hippocampus in the CUMS rats significantly increased (*p* < 0.05). The Ly294002 group showed a significantly reduced level of PI3K compared to the PI3K levels found in the CA1 region of the hippocampus of the Xiaoyaosan + Ly294002 group (*p* < 0.05). There was no difference between the rats in the vehicle (W) and vehicle (D) groups. The level of PI3K mRNA in rats of Xiaoyaosan + Ly294002 group tended to decrease compared to that of the Xiaoyaosan group. The western blot results (Figures 7D,E) were consistent with the immunohistochemistry results. These results indicate that Ly294002 blocked the expression of PI3K and that Xiaoyaosan significantly reversed the decrease in PI3K induced by CUMS. Compared to the rats in the Xiaoyaosan group, the expression of PI3K in the CA1 region of the hippocampus of the rats in the Xiaoyaosan + Ly294002 group was lower (*p* < 0.05).

**FIGURE 8:**
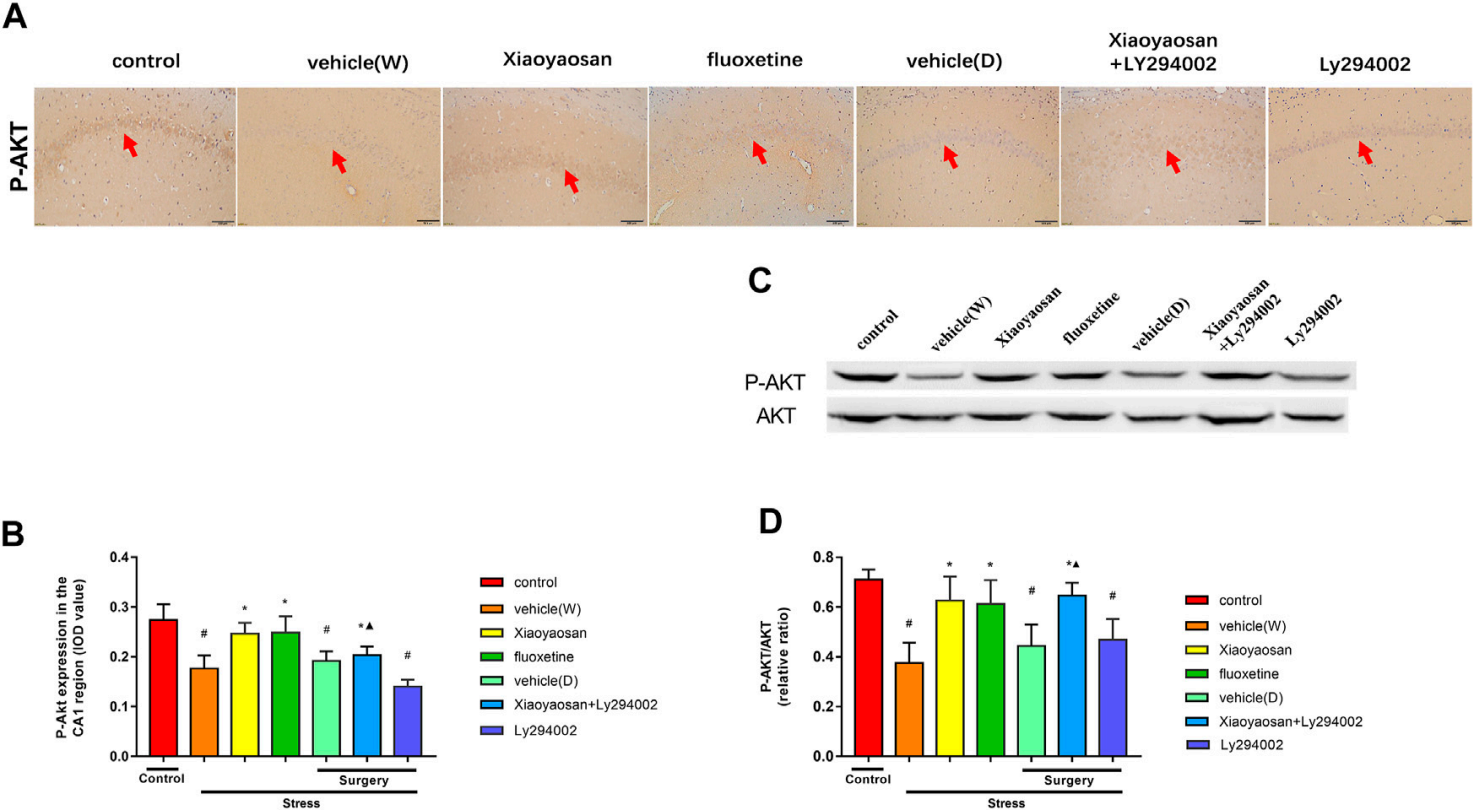
Xiaoyaosan elevates the expression of P-AKT/AKT in the hippocampal CA1 region of CUMS rats P-AKT/AKT in the hippocampal CA1 region of CUMS rats **(A)** Representative micrographs of immunohistochemical staining (sections were counterstained with hematoxylin; original magnifification, ×200) and **(B)** quantitative analysis showing the expression of P-AKT in the hippocampal CA1 region **(C)**. Representative images and western blot analysis **(D)** of western blot assay showing the relative expression ratio of P-AKT/AKT in the hippocampal CA1 region. All data are expressed as the mean ± SD. ^#^ *p* < 0.05 compared to the control group; ^*^ *p* < 0.05, compared to the vehicle (W) group; ^▲^ *p* < 0.05 compared to the Ly294002 group, *n* = 6.

As shown in Figures 8A,B, the expression of P-AKT in the rat hippocampal CA1 area in the vehicle (W), vehicle (D) and Ly294002 groups was lower than that in the control group (*p* < 0.05). Interestingly, upon the treatment with Xiaoyaosan, fluoxetine or Xiaoyaosan + Ly294002 for 3 weeks, the expression of P-AKT in the CA1 region of the hippocampus in the CUMS rats significantly increased (*p* < 0.05). Compared to that of the rats in the Ly294002 group, the expression of P-AKT in the hippocampal CA1 region of the rats in the Xiaoyaosan + Ly294002 group was higher (*p* < 0.05). As shown in Figures 8C,D, the expression of P-AKT/AKT ratio in the rat hippocampal CA1 area in the vehicle (W), vehicle (D) and Ly294002 groups was lower than that in the control group (*p* < 0.05). Upon treatment with Xiaoyaosan, fluoxetine or Xiaoyaosan + Ly294002 for 3 weeks, the expression of P-AKT/AKT ratio in the CA1 region of the hippocampus in the CUMS rats significantly increased (*p* < 0.05). Compared to that of the rats in the Ly294002 group, the expression of P-AKT/AKT ratio in the hippocampal CA1 region of the rats in the Xiaoyaosan + Ly294002 group was higher (*p* < 0.05).

## Discussion

In this current study, we found that CUMS rats showed downregulated NR2B expressions and thus excessive accumulations of glutamate in the hippocampal CA1 region and consequently led to the loss of MAP2 neurons, which could be reversed by the treatment of Xiaoyaosan. This shows that chronic stress led to the atrophy of neurons, and then triggers the development of depression, which may be related to the disorder of glutamate metabolism. In addition, the Xiaoyaosan treatment can exert the antidepressant effect by rescuing hippocampal neurons loss induced by the glutamate-mediated excitotoxicity in CUMS rats. Furthermore, we found that the neuronal marker MAP2 in hippocampus of CUMS rats was significantly increased after the administration of Xiaoyaosan, which depends on the PI3K/AKT signaling pathway verified by the block of the PI3K with Ly294402. From these results, we draw the conclusions that the effect of Xiaoyaosan is dependent on NR2B and the PI3K/AKT signaling pathway to alleviate the neuronal damages induced by glutamate.

Stress is closely related to neurological disorders. CUMS is a common rodent stress model that is frequently used in modern research investigating depression. (Willner and Mitchell, 2002; Wan et al., 2014). Katz and colleagues first reported the CUMS model (Katz et al., 1981). After years of study, CUMS has played an important role in the discovery and development programmes of antidepressant drugs. Our previous research demonstrated that 6 weeks of CUMS in rats produced obvious depression-like behavior (Li, 2018). Therefore, in this study, we established a 6-weeks CUMS rat model to observe the antidepressant effect and possible mechanism of Xiaoyaosan in CUMS rats. Appetite is an important index of depression-like behavior (Bersani et al., 2010). It has been reported that 48% of adult depressed patients have a decreased appetite. (Simmons et al., 2016). In this research, the food intake and body weight of the rats were detected each week. At 0 weeks, no significant difference was observed in food intake among the seven groups. After three weeks, in contrast with the rats in the control group, the food intake and body weight of the CUMS rats was significantly reduced. The food intake levels and body weights of the rats in the Xiaoyaosan and Xiaoyaosan + Ly294002 groups were increased, which was in contrast to the vehicle (W) group. Meanwhile, an OFT, SPT and FST were performed to assess depressive behavior. The OFT was used to quantitatively evaluate the rodents’ spontaneous activity exploration behavior and state of depression and anxiety (Wang et al., 2011a; Wang et al., 2011b). We found that the rats in the vehicle (W) group spent less time and traveled a shorter total distance than the rats in the control group. The Xiaoyaosan and Xiaoyaosan + Ly294002 groups showed significant improvement. The SPT is the gold standard for evaluating the degree of pleasure loss in rodents. Anhedonia is manifested by the lack of interest in a reward stimulus, which is a manifestation of affective disorders, including depression (Weiss, 1997; Belovicova et al., 2017). Therefore, an SPT was conducted on day 0 as the baseline to exclude depressed and anxious rats. In this study, the CUMS rats showed a lower degree of preference for sucrose after 3 weeks of CUMS. After the administration of Xiaoyaosan or Xiaoyaosan + Ly294002 for another 3 weeks, the preference for sucrose of the rats was significantly increased compared to that of the rats in the vehicle (W) group, indicating that Xiaoyaosan exhibited a good antidepressant performance and that Ly294002 may be an inhibitor of the pathway by which Xiaoyaosan plays an antidepressant role. The FST is commonly used to evaluate depression-like behavior in rodents (Belovicova et al., 2017). In the present research, we found that the immobility time of the rats in vehicle (W) group was significantly longer than that of the controls. The administration of Xiaoyaosan and Xiaoyaosan + Ly294002 decreased the immobility time. Fluoxetine has been reported to improve depression-like behavior in CUMS rats (Zhao et al., 2020) and is widely used in clinical treatment. Therefore, fluoxetine was used as a positive control drug in this study (Corrigan et al., 2000). The results of the behavior experiment showed that Xiaoyaosan could ameliorate CUMS depression-like behavior in rats.

Our current results revealed that Xiaoyaosan reduced the level of glutamate in hippocampal CA1 region of CUMS rats. It has been demonstrated that glutamate are the primary excitatory neurotransmitters with both intrinsic and extrinsic control of information flow in the brain. Evidence has proved that the abnormalities in the synthesis, metabolism, and reuptake into neurons of glutamate in the brains lead the pathophysiology of depression (Murrough et al., 2017). Further preclinical studies also report that acute and chronic stress procedures increase extracellular glutamate in the hippocampus, and this has led glutamate-mediated excitotoxicity via actions at extra synaptic N-methyl-D-aspartate receptors (NMDARS) are responsible for the loss of neurons in these brain regions (Autry et al., 2011).

MAP2 is a neuron-specific cytoskeletal protein used as a marker of neuronal phenotypes (Izant and McIntosh, 1980). Our study showed that the expression of MAP2 in the hippocampal CA1 region of the CUMS rats was reduced and that Xiaoyaosan reversed this change. This finding indicates that Xiaoyaosan can promote neuronal growth. The results of other studies are consistent with our results. According to the research by Soetanto et al., compared to healthy people, depressive patients’ hippocampi express lower levels of MAP2 (Soetanto et al., 2010). Abdel-Rahman et al. revealed that the expression of MAP2 in the cerebral cortex and the CA1 and CA3 regions of the hippocampus is reduced after 28 days of CUMS exposure (Abdel-Rahman et al., 2004). The IF staining showed that the expression levels of neuron MAP2 in the CUMS rats were lower (Yang et al., 2017). Xiaoyaosan also found an increase in the expression of the MAP2 protein in rats following corticosterone-induced stress injury to PHN (Cao et al., 2016). Serum containing Xiaoyaosan could correct the imbalance in the expression of NR2A/NR2B in the PHN of rats (Sun, 2010). Paeoniflorin (Zhang et al., 2015) and ferulic acid (Moghadam et al., 2018) are the active components of Xiaoyaosan and have been shown to increase MAP2 expression in damaged neurons. In addition, C. R. Yang et al. found that fluoxetine could increase the expression of MAP2 in the hippocampus (Yang et al., 2015). Therefore, fluoxetine was used as a positive control to observe neuronal growth in this experiment. However, further studies are still needed to determine the potential mechanism of the neuroprotective effect of Xiaoyaosan in CUMS rats.

The NMDAR family includes GluN1, GluN2 (NR2A, NR2B, NR2C, NR3D) and GluN3 (NR3BA and NR3B), which is important for controlling synaptic plasticity and memory function (Waxman and Lynch, 2005; Kim et al., 2011; Wang et al., 2011a; Wang et al., 2011b). NMDARs are also essential for the formation of neuronal circuits and the nervous system during development (Wang et al., 2014). Among the NMDAR family, NR2B was demonstrated to be the most strongly associated with depression (Duman and Voleti, 2012; Wang et al., 2014). In addition, the downregulation of NR2B expression can trigger PI3K pathway. So, in this present study, we detected the NR2B expression in the hippocampal CA1 region of CUMS rats. Jun Dong et al. found that the level of glutamate in the hippocampus of depressed rats was increased and that the expression of NR2B was decreased, which may cause depression (Dong et al., 2010). Wang et al. also found that NR2B expression was increased in the hippocampus of CIS rats (Wang et al., 2013). Yang et al. found that the expression of the NMDAR 2B subunit in the hippocampus of depression-like mice was decreased (Yang et al., 2018). As shown in this study, NR2B expression in the CUMS rats was lower than that in the control group. The glutamate level in CUMS rats is high (as shown in our previous study (Liu et al., 2019), and the excessive activation of NMDARs could be caused by high levels of glutamate. In addition, the accumulation of glutamate in association with a reduction in NR2B could aggravate depression. Sartorius et al. (Sartorius et al., 2007) also found that high glutamate contents and/or low glutamate aminobutyric acid (GABA) levels were correlated with depression. The accumulation of glutamate and downregulation of NR2B in animals with depression-like behavior could cause instability in the glutamate nervous system and activation of NR2B. Our study also indicated that the level of glutamate in CUMS rats was high and that the Xiaoyaosan and Xiaoyaosan + Ly294002 treatments decreased the level of glutamate. The increase in NR2B in the Xiaoyaosan and Xiaoyaosan + Ly294002 treatment groups showed that the changes in NR2B in the hippocampal CA1 region of the CUMS rats could be reversed to some extent by both Xiaoyaosan treatments. Moreover, paeoniflorin (Mao et al., 2010), liquiritin (Teng et al., 2014) and liquiritigenin (Yang et al., 2013), which are three major components in Xiaoyaosan, have been previously shown to protect against glutamate-induced neurotoxicity in PC12 cells and HT22 cells. Altogether, these findings may indicate that the antidepressant effect of Xiaoyaosan in CUMS rats is driven by the improved expression of NR2B, resulting in a reduced glutamate content and enhanced neuronal growth.

Then, in order to further explore how levels of NR2B to cause the loss of neurons in CUMS rats and how Xiaoyaosan works, we further examined the changes in the PI3K-AKT signaling pathway, an important downstream signaling pathway of NR2B. It has been found that the activation of PI3K-AKT signaling pathway act as a major regulator in the process of neuronal survival against death induced by various stimuli. (Dai et al., 2016). Especially, the phosphorylation of Akt could protect the mitochondrial integrity and function, and thereby inhibits neuronal apoptosis (Jazvinscak et al., 2018). The present study shows that Xiaoyaosan increased the expression of PI3K and the P-AKT/AKT ratio in CUMS rats. Akt is the first downstream target modulated by the activation of the PI3K/Akt pathway. CUMS was found to decrease the levels of hippocampal PI3K and AKT (Yang et al., 2017). The expression of P-Akt in the hippocampus of sodium arsenite-exposed rats was significantly lower than that in the control group (Srivastava et al., 2018). Naijun Yuan et al. also found that Xiaoyaosan could increase the P-AKT/AKT ratio in cortisol-induced PC-12 cells (Yuan et al., 2020). Decreased Akt activity and decreased extracellular regulatory kinase activity coexist in depression, and autopsy studies have demonstrated that the activity of the PI3K-Akt signaling pathway is decreased in suicidal patients with major depression (Karege et al., 2011). In addition, paeoniflorin, which is a major component in Xiaoyaosan, has been found to promote P-Akt expression in depressed rats (Chen et al., 2015). Interestingly, the PI3K/Akt pathway could be induced by NMDAR activation in depression (Brennan-Minnella et al., 2013). In addition, the increased levels of NR2B and P-Akt had an overall positive effect on the survival of neurons in the CA1 region of the hippocampus (Dai et al., 2016). We sought to determine whether Xiaoyaosan can activate the downstream PI3K/AKT signaling pathway after upregulating NR2B expression in the hippocampal CA1 region. Therefore, the PI3K inhibitor Ly294002 (Shen et al., 2019) was added to the Xiaoyaosan treatment. Our study showed that Xiaoyaosan and Xiaoyaosan + Ly294002 could increase the expression of PI3K and the P-AKT/AKT ratio. These results indicate that Xiaoyaosan may reverse the blocking effect of the Ly294002 inhibitor and activate the PI3K/AKT pathway.

In this study, we found that the neuronal marker MAP2 in hippocampus of CUMS rats was significantly increased after the administration of Xiaoyaosan, which depends on the PI3K/AKT signaling pathway verified by the block of the PI3K with Ly294402. From these results, we draw the conclusions that the effect of Xiaoyaosan is dependent on NR2B and the PI3K/AKT signaling pathway to alleviate the neuronal damages induced by glutamate.

## Data Availability

The original contributions presented in the study are included in the article/Supplementary Material, further inquiries can be directed to the corresponding authors.

## Abbreviations

AKT Protein kinase B CIS Chronic immobilization stress CORT Corticosterone CUMS Chronic unpredictable mild stress GABA Glutamate aminobutyric acid HAMD Hamilton depression rating scale HPLC High‐performance liquid chromatography IHC Immunohistochemical IF Immunofluorescence LTP Long‐term potentiation MAP2 Microtubule‐associated protein 2 NMDA N‐methyl‐d‐aspartate NR2B N‐methyl‐D‐aspartate receptor subtype 2B NMDAR N‐methyl‐D‐aspartate receptor PHN Primary hippocampal neurons PI3K Phosphoinositide 3‐kinase RT‐qPCR Quantitative real‐time polymerase chain reaction SDS Self‐rating Depression Scale TCM Traditional Chinese medicine
