# Corrigendum: Xiaoyaosan alleviates hippocampal glutamate-induced toxicity in the CUMS rats via NR2B and PI3K/Akt signaling pathway

**DOI:** 10.3389/fphar.2022.1042762

**Published:** 2022-12-15

**Authors:** Xueming Zhou, Chenyue Liu, Yueyun Liu, Qingyu Ma, Xin Zhao, Youming Jiang, Xiaojuan Li, Jia-Xu Chen

**Affiliations:** ^1^ School of Traditional Chinese Medicine, Beijing University of Chinese Medicine, Beijing, China; ^2^ School of Basic Medical Sciences, Heilongjiang University of Chinese Medicine, Haerbin, China; ^3^ Formula-pattern Research Center, School of Traditional Chinese Medicine, Jinan University, Guangzhou, China

**Keywords:** depression, xiaoyaosan, NR2B, PI3K/Akt pathway, hippocampal CA1 region, glutamate

In the published article, there was an error in Figures 5A, 8A as published. The wrong images were used in Figure 5A at the vehicle (W) and vehicle (D) group as well as Figure 8A. The correct version of the figure shows below. The corrected Figures 5, 8 and its caption appear below.

**FIGURE 5:**
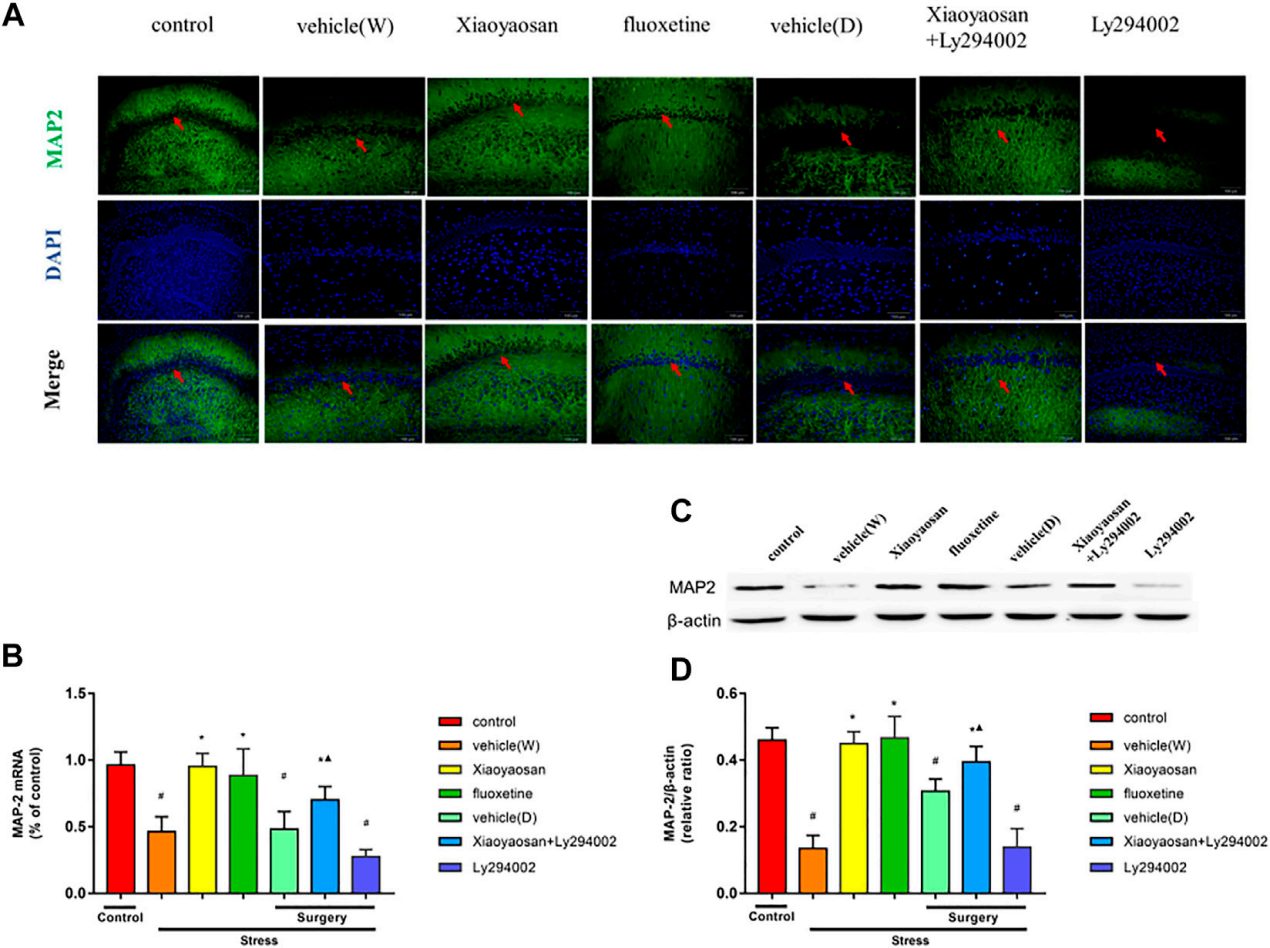
Xiaoyaosan elevates the expression of MAP2 in the hippocampal CA1 region of CUMS rats (**A**, original magnifification, ×200) Xiaoyaosan and Xiaoyaosan + Ly294002 promoted MAP2 expression in the CUMS rats. The green color represents MAP2 staining, and the blue color represents nuclear staining **(C)** Representative images and western blot analysis **(D)** of western blot assay showing the relative expression of MAP2 **(B)** Level of MAP2 mRNA in the hippocampal CA1 area of the rats in the treatment and control groups. All data are expressed as the mean ± SD. ^#^ *p* < 0.05 compared to the control group; ^*^ *p* < 0.05 compared to the vehicle (W) group; ^▲^ *p* < 0.05 compared to the Ly294002 group, *n* = 6.

**FIGURE 8:**
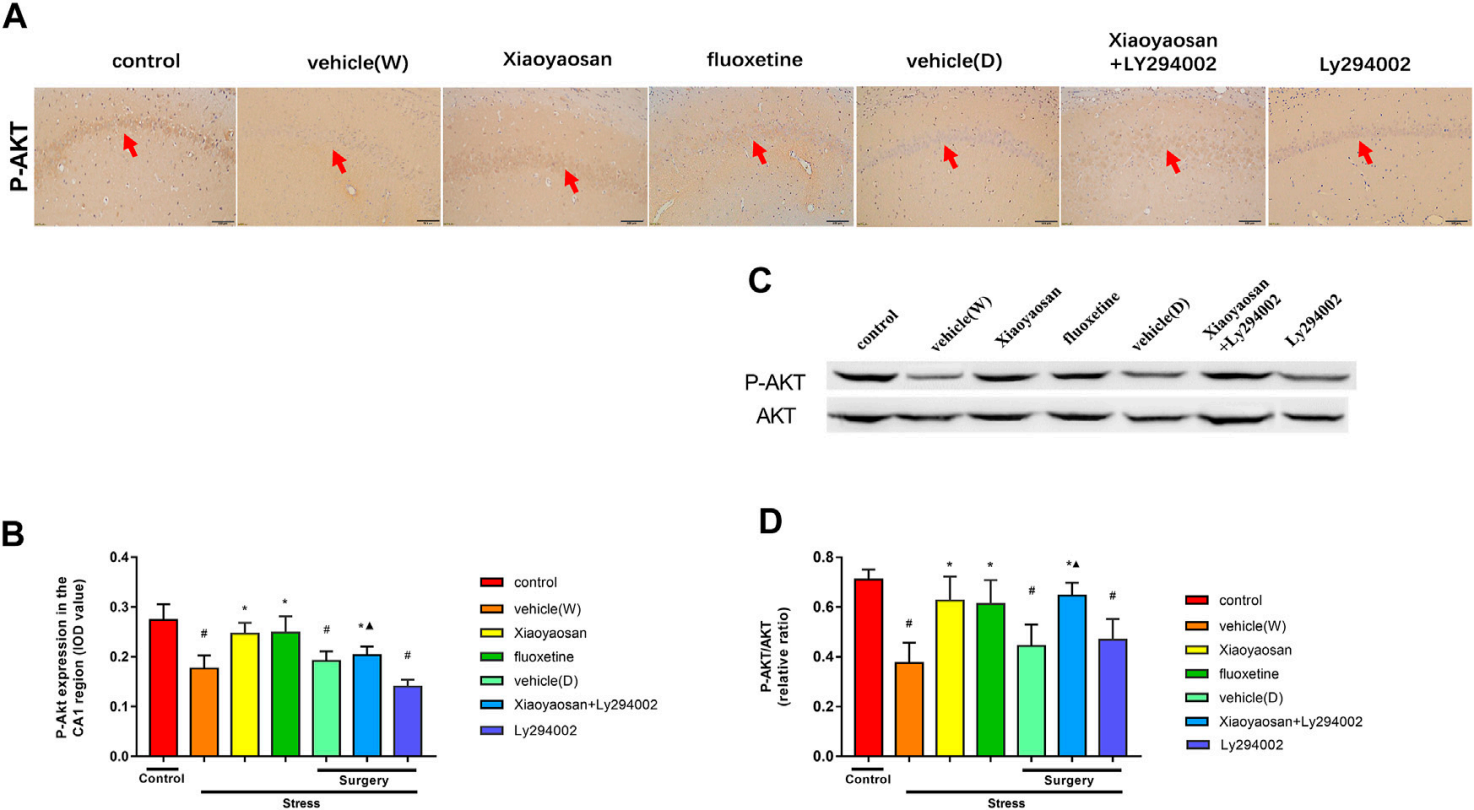
Xiaoyaosan elevates the expression of P-AKT/AKT in the hippocampal CA1 region of CUMS rats P-AKT/AKT in the hippocampal CA1 region of CUMS rats **(A)** Representative micrographs of immunohistochemical staining (sections were counterstained with hematoxylin; original magnifification, ×200) and **(B)** quantitative analysis showing the expression of P-AKT in the hippocampal CA1 region **(C)**. Representative images and western blot analysis **(D)** of western blot assay showing the relative expression ratio of P-AKT/AKT in the hippocampal CA1 region. All data are expressed as the mean ± SD. ^#^ *p* < 0.05 compared to the control group; ^*^ *p* < 0.05, compared to the vehicle (W) group; ^▲^ *p* < 0.05 compared to the Ly294002 group, *n* = 6.

The authors apologize for this error and state that this does not change the scientific conclusions of the article in any way. The original article has been updated.

