# Supplementary figures and images for: Xiaoyaosan Alleviates Hippocampal Glutamate-Induced Toxicity in the CUMS Rats via NR2B and PI3K/Akt Signaling Pathway
Source: Front Pharmacol. 2021 Apr 12;12:586788. doi: 10.3389/fphar.2021.586788 (PMC8075411; doi:10.3389/fphar.2021.586788)

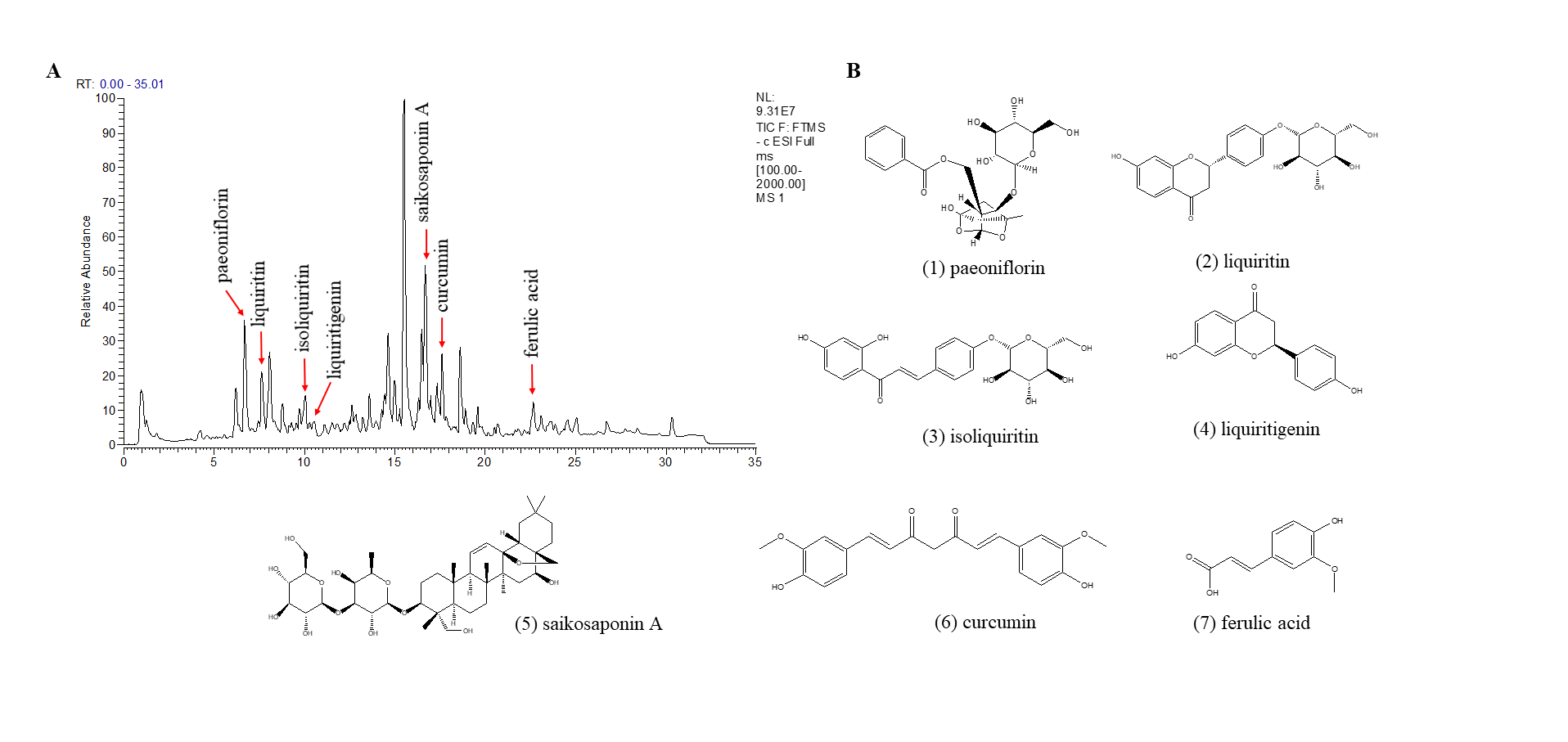

Supplement: Supplementary file 1 [file Image1.TIF]
